# Optimization of neural-network model using a meta-heuristic algorithm for the estimation of dynamic Poisson’s ratio of selected rock types

**DOI:** 10.1038/s41598-023-38163-0

**Published:** 2023-07-08

**Authors:** Umer Waqas, Muhammad Farooq Ahmed, Hafiz Muhammad Awais Rashid, Mohamed Ezzat Al-Atroush

**Affiliations:** 1 Department of Geological Engineering, University of Engineering and Technology, Lahore, 54890 Pakistan; 2 Department of Engineering Management, College of Engineering, Prince Sultan University, Riyadh, 11543 Saudi Arabia

**Keywords:** Engineering, Civil engineering

## Abstract

This research focuses on the predictive modeling between rocks' dynamic properties and the optimization of neural network models. For this purpose, the rocks' dynamic properties were measured in terms of quality factor (Q), resonance frequency (FR), acoustic impedance (Z), oscillation decay factor (α), and dynamic Poisson’s ratio (*v*). Rock samples were tested in both longitudinal and torsion modes. Their ratios were taken to reduce data variability and make them dimensionless for analysis. Results showed that with the increase in excitation frequencies, the stiffness of the rocks got increased because of the plastic deformation of pre-existing cracks and then started to decrease due to the development of new microcracks. After the evaluation of the rocks’ dynamic behavior, the *v* was estimated by the prediction modeling. Overall, 15 models were developed by using the backpropagation neural network algorithms including feed-forward, cascade-forward, and Elman. Among all models, the feed-forward model with 40 neurons was considered as best one due to its comparatively good performance in the learning and validation phases. The value of the coefficient of determination (R^2^ = 0.797) for the feed-forward model was found higher than the rest of the models. To further improve its quality, the model was optimized using the meta-heuristic algorithm (i.e. particle swarm optimizer). The optimizer ameliorated its R^2^ values from 0.797 to 0.954. The outcomes of this study exhibit the effective utilization of a meta-heuristic algorithm to improve model quality that can be used as a reference to solve several problems regarding data modeling, pattern recognition, data classification, etc.

## Introduction

Rock dynamics has a wide range of applications in many rock engineering-related projects including tunneling and excavation, reservoir modeling, hydrocarbon reserve estimation, geotechnical earthquake engineering, drilling and blasting, rock fragmentation processes, and subsurface imaging^1–4^. The knowledge of rock dynamics provides a valuable set of information to discern the engineering response of rocks subjected to dynamic loading conditions. Seismic events, shock vibrations, and impact loads are common sources of dynamic loadings that affect particles' displacement, velocity, and acceleration^5^. Repeated loading cycles or an increasing loading rate considerably alter the mechanical characteristics of rocks. The rocks are not perfectly elastic materials rather they are brittle, anisotropic, discontinuous, and heterogeneous. Therefore, the induced strains in rocks are not fully recoverable even in the elastic domain^3, 6^. Over the period, the coalescence of these micro strains may lead to major deformation or failure.

The Poisson’s ratio is one of those important parameters that play a significant role in the design work of underground structures such as the foundation of megastructures, radioactive waste repositories, tunnels, large caverns, etc.^7–9^. It is the ratio of lateral strain to the axial strain of a rock specimen under static or dynamic loading^10^. The Poisson’s ratio may be dynamic or static depending on the state of stress. The dynamic Poisson’s ratio is determined by testing rock specimens non-destructively as per the standard procedure described by the American Society for Testing and Materials (ASTM C215^11^) to measure their acoustic wave velocities or resonance frequencies. These parameters are further utilized to find the dynamic Poisson's ratio. On the other hand, to measure static Poisson’s ratio rock samples are tested destructively as per the ASTM D3148^12^.

In underground excavation, the subsurface imaging and determination of dynamic properties (specifically dynamic elastic moduli, dynamic Poisson’s ratio, acoustic impedance, quality factor, etc.) considerably help to ascertain the dynamic characteristics of rocks^5^. Generally, the dynamic Poisson’s ratio shows higher values than the static one because rocks are considered more sensitive toward dynamic loading^13^. The incongruity between the static-dynamic Poisson’s ratio is attributed to several factors such as rock lithology, mineral composition, loading conditions, strain rate, strain amplitude, etc.^14–16^.

Determination of dynamic Poisson’s ratio either in the field or laboratory needs calibrated instruments and good expertise^15^. Apart from the experimentation, dynamic Poisson’s ratio can be estimated by empirical relationships. Researchers have developed several empirical relationships based on rock density, strength, acoustic wave velocities, porosity, permeability, and elastic moduli^15, 17–22^.

The statistical models generate varying outputs for a single parameter due to two major reasons: the dependent variable is regressed with a different set of predictors, and the input variables in the empirical equations employ mean values that often lead to underestimation or overestimation of the outputs^23, 24^. In conventional statistical analysis, multicollinearity and overfitting significantly affect the prediction ability of multiple linear-nonlinear regression models. Apart from these traditional approaches, the use of artificial intelligence and machine learning algorithms gives satisfactory outcomes.

Unlike regression analysis, neural network-based prediction models do not consider mean values rather they use data variance. Thus, they provide optimal solutions for both linear and nonlinear problems of experimentally measured data. Recently published studies have proposed different models for the estimation of Poisson’s ratio. Zhang and Bentley in 2005^15^ studied the static-dynamic behavior of clastic rocks and determined Poisson’s ratio from two independent factors: solid rock and dry or wet cracks. Al-anazi et al. 2011^25^ used an alternating conditional expectation algorithm to predict Poisson’s ratio from measured data of porosity, pore pressure, bulk density, compressional, and shear wave travel time. Asoodeh in 2013^26^ estimated the Poisson’s ratio from conventional well-log data using a radial basis neural network, Sugeno fuzzy inference system, neuro-fuzzy algorithm, and simple averaging method. He used the outputs obtained from each expert to construct a committee machine with an intelligent system using a hybrid genetic algorithm-pattern search technique. The integrated outcomes were much better than the output of the individual one. Abdulraheem in 2019^21^ performed a neural network and fuzzy logic type-2 analysis to estimate Poisson’s ratio of rocks. He found that the neural network model had better prediction ability than the fuzzy logic model.

In light of the above discussion, it is evident that the artificial neural network has a competitive edge over conventional statistical modeling techniques. In regression analysis, highly correlated predictors produce multicollinearity and overfitting that’s why such input variables are excluded from the empirical equations to overcome dimensionality problems^16^. The artificial neural network can predict target values with minimum estimated residuals and can easily control the aforementioned issues without excluding a single input variable. The neural network can accommodate several rock properties as input variables to completely define its characteristics. Apart from its advantages, it has the demerits of a slow learning rate and getting trapped in local minima^18^. The training and learning of neural networks with a powerful optimizer such as particle swarm optimization (PSO) overwhelm these problems and ameliorates the performance of neural network models. The PSO is the population-based stochastic optimization approach that works on the social behavior of bird flocks or fish schools and has a powerful ability to solve continuous or discrete optimization problems^27^. In comparison with other optimizers, PSO has a less complex structure and simple parameter relationships^28^.

This study measures the dynamic properties of rocks especially Poisson’s ratio using the cyclic excitation frequency method. This approach has not been addressed comprehensively in the literature. In addition, several attempts have been made to develop predictive relationships for different physic-mechanical parameters of rocks such as compressive strength, tensile strength, shear strength, elastic moduli, porosity, permeability, acoustic wave velocities, etc.^16, 29–32^. There is limited literature available that focuses on the predictive modeling of dynamic Poisson’s ratio. Apart from the conventional modeling techniques, this research favors using a gradient descent-free optimizer such as PSO for the training of a selected neural network model.

The first objective of this study is to anticipate the dynamic behavior of selected sedimentary rocks in terms of their quality factor, resonance frequency, oscillation decay factor, acoustic impedance, and Poisson's ratio. Secondly, this research develops neural network models for the estimation of dynamic Poisson’s ratio using 3 neural network algorithms including feed-forward, cascade-forward, and Elman. The third objective is to train the proposed neural network model with particle swarm optimization to enhance model performance. The outcomes of this study can be used as a reference to solve the problems related to data modeling, prediction analysis, and indirect estimation of rock properties.

## Materials and methods

The representative rock boulders of sandstone, limestone, dolomite, and marl were obtained from their outcrop exposed in the eastern part of the Salt Range, Punjab, Pakistan. The stratigraphic sequence, geological age, characteristics, and features of these rock units are described in Table 1. The collected rock boulders were free from major discontinuities and carried into the laboratory to prepare the required number of core specimens. A total of 50 NX-size (i.e., length to diameter ratio of 2 to 2.5 with a diameter of 54.7 mm) rock core samples were prepared and put into the desiccator to minimize the effect of moisture content on their dynamic properties.

**Table 1:** The characteristics and features of the selected sedimentary rock types.

Geological age	Rock formation	Rock unit	Characteristics
Precambrian	Salt range	Marl	It is a reddish-orange color gypsiferous soft rock with an ample amount of clay minerals. It contains mica, iron oxide, and variable sizes of quartz crystals. It is abundantly found in the eastern part of the Salt Range
Cambrian	Khewra	Upper sandstone	It is a Cambrian aged purple to yellowish-brown color medium grain thickly bedded sandstone. It preserves a sufficient number of vertebrate fossils. Based on the strength characteristics and mineral composition, it has been divided into 3 rock units. It contains medium grain quartz, feldspar, mica, and a trace amount of clay minerals
		Middle sandstone	
		Lower sandstone	
Cambrian	Kussak	Sandstone	It is a glauconitic greenish-grey color sandstone that preserves 2–10 inches long thin lenses of fossils. The oolitic erinaceous dolomite and inter-bedded conglomerate are the other prominent units of this formation
Cambrian	Jutana	Dolomite	The dolomite in this formation is divided into two distinct zones. The upper exposed unit is a light green to dirty-white color dolomite; whereas, the lower unit is massive sandy dolomite with breccia inclusions. It contains an 80–90% dolomite mineral. The thickness of its bed varies from 50 to 80 m
Permian	Tobra	Sandstone	It is an off-white color medium to coarse grain thickly bedded sandstone that is comprised of facies above a major unconformity. Its primary minerals are quartz, feldspar, mica, and iron oxide. It shows signs of metamorphism as well. The conglomerate unit caps this Permian succession
Eocene	Namal	Limestone	The tertiary succession is composed of Namal and Sakesar formations. The Namal formation mainly consists of a thinly bedded, laminated, fine grain, and yellowish-white color limestone. It has an abundant number of fossils i.e. foraminifera. Its thickness varies from 70 to 300 m in the Salt Range region. The limestone of Sakesar formation is comprised of massive and nodular facies. The upper unit is an off-white color nodular limestone characterized by the chert lenses. The lower unit is a light-grey color massive limestone that preserves marine fossils. In the Salt Range, its thickness varies from 30 to 130 m. Their primary minerals are dolomite, calcite, and micrite
Eocene	Sakesar	Massive limestone	
		Nodular limestone	

### Laboratory testing

**Figure 1:**
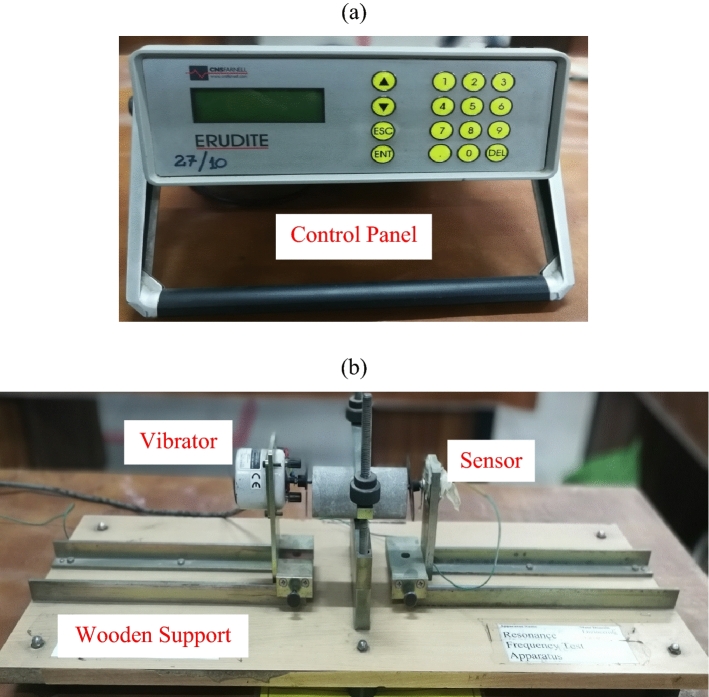
Experimental setup for the testing of rock core samples.

A series of non-destructive tests were conducted on the rock core samples to measure their dynamic properties using Erudite Resonance Frequency Meter as per the ASTM C-215 standard. This instrument mainly consists of a vibrator, receiver, and control panel as shown in Fig. 1. In order to measure the dynamic properties of rocks, the applied loading frequency of 7–16 kHz was allowed to pass through the rock core samples clamped between the vibrator and receiver. The rock specimens were tested in longitudinal mode and torsion mode to measure their resonance frequency and quality factor in each case. The measured resonance frequency was used to determine elastic moduli and oscillation decay factor. Whereas elastic moduli were further used to find acoustic impedance and Poisson’s ratio. The experimentally acquired dataset of rock dynamic properties is shown in Table 2. Equations (1), (2) and (3) are described in the ASTM C215^11^ testing standard. Whereas Eqs. (4), (5) and (6) are explained by Kramer 1996^6^ for earthen materials. Mathematically, these parameters can be expressed as follows:

1 $${Q}_{P}=\frac{{FR}_{P}}{Bandwidh}\, OR \,{Q}_{S}=\frac{{FR}_{S}}{Bandwidh}$$

2 $$E={D}_{cylinder}W{({FR}_{P})}^{2}$$

3 $$G={B}_{cylinder}W{({FR}_{S})}^{2}$$

4 $$v=\frac{E}{2G}-1$$

5 $${\alpha }_{P}=\frac{\pi \,{FR}_{P}}{{Q}_{P}} \,OR\, {\alpha }_{S}=\frac{\pi {FR}_{S}}{{Q}_{S}}$$

6 $${Z}_{P}=\sqrt{E\, \rho }\, OR\, {Z}_{S}=\sqrt{G\, \rho }$$

where: The subscript P and S with the parameters denote their measured values in longitudinal mode and torsion mode respectively. Q is the quality factor, FR is the resonance frequency, Bandwidth is the difference between corner frequencies, E is the modulus of elasticity, D_cylinder_ is the constant and equal to 520 L/d^2^ with L and d are the sample length and diameter respectively, G is the modulus of rigidity, B_cylinder_ is the constant and equal to 400 L/A with L and A are the sample length and cross-section area respectively, W is the weight of the specimen, *v* is the dynamic Poisson’s ratio, α is the oscillation decay factor, Z is the acoustic impedance, and $\rho$ is the sample density.

**Table 2:** Experimentally measured dynamic properties of selected sedimentary rocks.

Rock unit	No. of samples	No. of tests	Q_P_	FR_P_ (kHz)	α_P_ (kHz)	Z_P_ (MPa*sec/m)	Q_S_	FR_S_ (kHz)	α_S_ (kHz)	Z_S_ (MPa*sec/m)	*v*
Salt Range Marl	5	10	20.63 ± 1.11	1.56 ± 0.01	0.24 ± 0.01	10.88 ± 0.06	17.36 ± 1.59	1.02 ± 0.01	0.19 ± 0.02	7.12 ± 0.06	0.17 ± 0.01
Upper Khewra Sandstone	5	10	16.68 ± 0.67	1.76 ± 0.03	0.33 ± 0.02	11.93 ± 0.23	11.68 ± 0.87	1.15 ± 0.02	0.31 ± 0.02	7.81 ± 0.11	0.16 ± 0.06
Middle Khewra sandstone	5	10	20.15 ± 0.91	1.64 ± 0.04	0.26 ± 0.02	10.00 ± 0.23	11.54 ± 3.06	1.05 ± 0.03	0.30 ± 0.07	6.42 ± 0.19	0.22 ± 0.03
Lower Khewra sandstone	5	10	22.00 ± 1.15	1.66 ± 0.02	0.23 ± 0.01	9.80 ± 0.12	16.90 ± 0.76	1.07 ± 0.01	0.20 ± 0.01	6.29 ± 0.06	0.21 ± 0.04
Kussak sandstone	5	10	22.27 ± 1.08	1.77 ± 0.01	0.25 ± 0.01	12.76 ± 0.08	16.05 ± 1.25	1.14 ± 0.01	0.22 ± 0.02	8.26 ± 0.04	0.19 ± 0.01
Jutana dolomite	5	10	21.67 ± 0.78	2.01 ± 0.02	0.29 ± 0.01	18.04 ± 0.15	14.75 ± 1.96	1.32 ± 0.01	0.28 ± 0.03	11.81 ± 0.06	0.17 ± 0.03
Tobra sandstone	5	10	22.23 ± 4.51	1.79 ± 0.04	0.26 ± 0.05	10.88 ± 0.23	10.73 ± 1.03	1.16 ± 0.03	0.34 ± 0.03	7.08 ± 0.15	0.18 ± 0.02
Namal limestone	5	10	24.86 ± 1.10	2.09 ± 0.09	0.27 ± 0.02	13.43 ± 0.55	14.92 ± 0.75	1.35 ± 0.05	0.28 ± 0.02	8.64 ± 0.30	0.21 ± 0.04
Sakesar massive limestone	5	10	27.87 ± 1.10	2.68 ± 0.03	0.30 ± 0.01	20.60 ± 0.21	16.95 ± 0.75	1.76 ± 0.03	0.33 ± 0.01	13.52 ± 0.26	0.16 ± 0.03
Sakesar nodular limestone	5	10	26.86 ± 1.10	2.03 ± 0.01	0.24 ± 0.01	14.62 ± 0.04	16.02 ± 0.85	1.31 ± 0.01	0.26 ± 0.01	9.42 ± 0.03	0.20 ± 0.01

The subscript P and S with the parameters denote the measured dynamic properties in the longitudinal mode and torsion mode, respectively.

After the experimentation, the acquired dataset was made dimensionless by simply dividing the parameter values measured in the longitudinal and torsion modes. The whole dataset was split into a training dataset (80% of the total population of the data) and a testing dataset (the remaining 20% of the total population of the data). The training dataset was fed to neural network algorithms (i.e. feed-forward, cascade-forward, and Elman) to estimate dynamic Poisson’s ratio through prediction modeling. For this purpose, MATLAB coding was used to execute the designed neural network system. The developed prediction models were validated by an additional validating dataset. The best-performing model was selected and optimized using a particle swarm optimizer to further ameliorate it.

The neural network model works in two phases: (1) the configuration phase and (2) the training phase. In the configuration phase, a model is designed stochastically after selecting the number of neurons, hidden layers, number of variables, weights, and biases. The model is run, and its outcomes are compared with the target data. In case of failure, the model is tuned parametrically or architecturally, until desired results are obtained. In architectural tuning, the structure of the network is modified by varying the number of neurons, hidden layers, etc. Whereas, in parametric tuning weights or biases are changed for network modification. In this research architectural modification was carried out by varying the number of neurons between 10 and 50 and keeping hidden layers constant (i.e., only 2 hidden layers). Before selecting the best one, models must be validated by experimentally acquired data. After selecting the model, the training or optimization phase starts. An optimizer is selected based on its learning rate and resistance to getting trapped in local minima. Almost all gradient descent optimizers face this issue, that’s why a gradient descent free meta-heuristic optimizer (i.e., PSO) was selected in this study.

To get some idea regarding the neural network algorithms and particle swarm optimization, a brief discussion as follows:

### Artificial neural network

**Figure 2:**
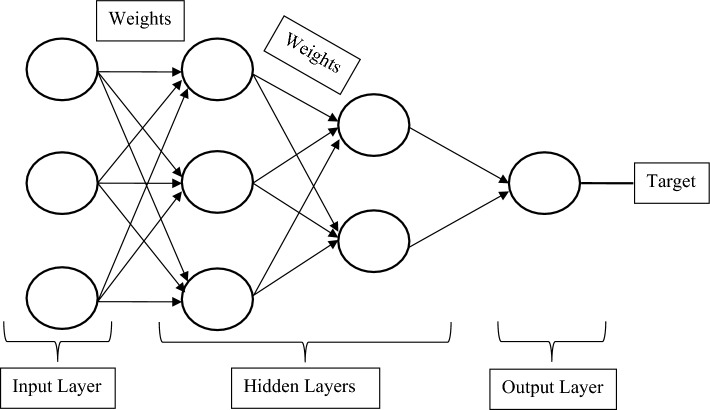
The generalized architecture of a neural network.

A neural network is a web of neurons that works on the principle of human brain intelligence^33, 34^. The artificial neurons or nodes are the core processing units of this adaptive system. The neural network is analogous to the biological neural network and nowadays is getting popularity to solve various complex problems related to artificial intelligence. There are several applications of neural networks in regression analysis, time series forecasting, signal processing, pattern recognition, decision-making, etc.^35, 36^. The performance of a neural network model is attributed to the design of its architecture. The simple architecture of the neural network is comprised of the input layer and output layer of the neurons connected with the synaptic weights. One or more hidden layers can be introduced between the input layer and the output layer to enhance its performance as shown in Fig. 2. The positive and negative values of the estimated synaptic weights reflect excitatory and inhibitory connections respectively. The input layer receives the input data, processes it, and transfers signals to the hidden layers with the estimated weights and biases. Most of the computations are done at hidden layers. Finally, the output layer gets weighted signals from hidden layers, processes them, and checks whether the estimated scores are close to the target data or not. If the estimated values are not within the defined constraints or threshold limits, then signals are sent back for their recalculation. At this stage, the applied training function adjusts the weights and minimizes the residuals between the target and output data.

**Figure 3:**
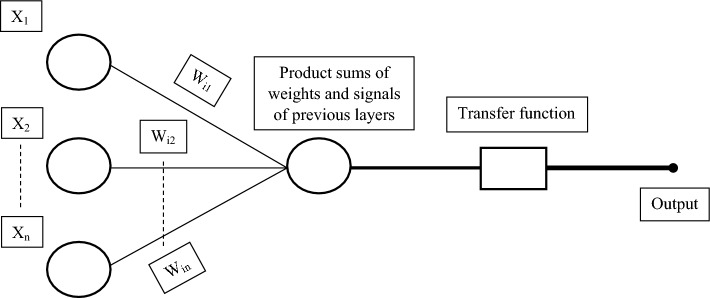
The basic architecture of an artificial neuron. Where X_1_ to X_n_ are input signals and W_i1_ to W_in_ are synaptic weights.

In an artificial neural network, two important points need to be addressed: (1) the selection of neurons in the hidden layer and (2) a suitable activation function. An inadequate number of neurons poorly fit the model on complex data; conversely, too many neurons overfit the model on data. In a neural network model, the number of neurons in the hidden layers is selected by the hit-and-trial method because no universal method has been developed yet that can provide guidelines in this regard^37^. The activation function defines the output of a node that gets the input or a set of input values. The purelin and poslin are considered linear transfer functions. Whereas, sigmoid and tangent hyperbola are taken as nonlinear transfer functions. In most cases, nonlinear transfer functions are preferred because of their better performance. The basic structure of an artificial neuron is described in Fig. 3.

### Feed-forward neural network

The feed-forward neural network is one of the early invented simple neural networks that has been widely used for regression and classification purposes^38^. In this hierarchical neural network, the connections between the signal processing units are free from any loop or cycle. Unlike their counterpart recurrent neural networks, it transfers the information only in a forward direction from the input layer to the output layer through the hidden layer (if any). A feed-forward neural network with no hidden nodes is known as a single-layer perceptron (SLP). Whereas, a feed-forward neural network supported with one or more hidden layers is called a multi-layer perceptron (MLP). Mathematically, it can be expressed as follows:

Set the target output values for the input nodes in layer L_o_

7 $$\mathrm{X}=\left\{{\mathrm{X}}_{1},\dots \dots \dots {\mathrm{X}}_{\mathrm{n}}\right\}\mathrm{ i}.\mathrm{e}.\, {\mathrm{O}}_{\mathrm{i}}^{0}={\mathrm{X}}_{\mathrm{i}}$$

Calculate the product sum of synaptic weights and the output of the previous layer at each node. The node outputs in a layer are considered input signals for the subsequent layer.

8 $${\mathrm{H}}_{\mathrm{i}}^{\mathrm{n}}=\sum_{\mathrm{l}=1}^{{\mathrm{r}}_{\mathrm{n}-1}}{\mathrm{W}}_{\mathrm{li}}^{\mathrm{n}}{\mathrm{O}}_{\mathrm{l}}^{\mathrm{n}-1}+{\mathrm{B}}_{\mathrm{i}}^{\mathrm{n}}\mathrm{\, for\, i}=1,\dots \dots .{\mathrm{r}}_{\mathrm{n}}$$

9 $${\mathrm{O}}_{\mathrm{i}}^{\mathrm{n}}={\upvarphi }({\mathrm{H}}_{\mathrm{i}}^{\mathrm{n}})\mathrm{\, for\, i}=1,\dots \dots .{\mathrm{r}}_{\mathrm{n}}$$

Compute the output for the output layer L_k_.

10 $${\mathrm{H}}_{1}^{\mathrm{k}}=\sum_{\mathrm{l}=1}^{{\mathrm{r}}_{\mathrm{k}-1}}{\mathrm{W}}_{\mathrm{l}1}^{\mathrm{n}}{\mathrm{O}}_{\mathrm{l}}^{\mathrm{n}-1}+{\mathrm{B}}_{1}^{\mathrm{k}}\mathrm{\, for\, i}=1$$

11 $${\mathrm{O}}_{1}^{\mathrm{k}}={\upvarphi }_{\mathrm{o}}({\mathrm{H}}_{1}^{\mathrm{k}})\mathrm{ \,for\, i}=1,$$

where: ${\mathrm{H}}_{\mathrm{i}}^{\mathrm{n}}$ is the product sum of weights and previous layer node outputs along with bias ${\mathrm{B}}_{\mathrm{i}}^{\mathrm{n}}$ for ith perceptron in the layer L_n_, ${\mathrm{W}}_{\mathrm{li}}^{\mathrm{n}}$ is the weight for ith perceptron in layer L_n_ connected with the lth node in the layer L_n-1_, ${\mathrm{O}}_{\mathrm{i}}^{\mathrm{n}}$ is the output for ith perceptron in the layer L_n,_ ${\mathrm{r}}_{\mathrm{n}}$ is the number of nodes in the layer L_n_, and $\mathrm{\varphi }$ is the transfer function.

### Cascade-forward neural network

**Figure 4:**
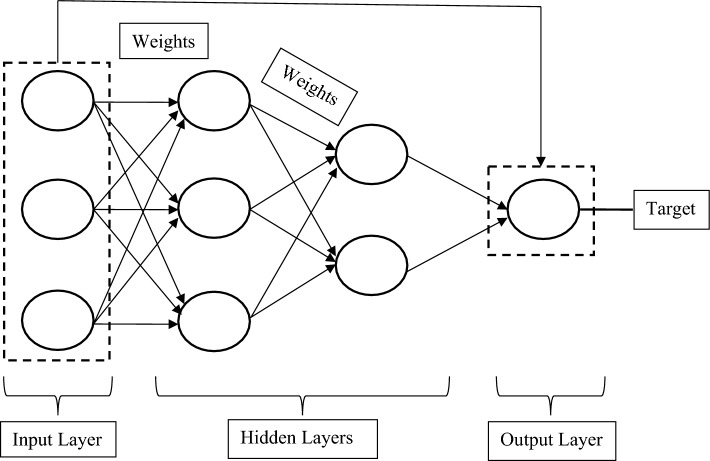
The basic architecture of a cascade-forward neural network model.

The cascade-forward neural network is a modified form of a feed-forward neural network that works similarly to its parent feed-forward algorithm. In the cascade-forward models, the input layer is connected to all subsequent layers to get better results. For example, in a three-layer cascade-forward model, the input layer is connected to both the hidden layer and output layer as shown in Fig. 4. This additional connection improves the learning rate of the model to obtain the required outputs with minimum computational time. The nonlinear and linear activation functions are applied to the hidden layers and an output layer respectively to reach the optimized status. The generalized mathematical expression can be expressed as follows:

12 $$\mathrm{O}={\mathrm{\varphi }}_{\mathrm{i}}\left(\sum_{\mathrm{i}=1}^{\mathrm{n}}{\mathrm{W}}_{\mathrm{i}}^{\mathrm{n}}{\mathrm{X}}_{\mathrm{i}}\right)+{\mathrm{\varphi }}_{\mathrm{o}}\left[\sum_{\mathrm{l}=1}^{\mathrm{k}}{\mathrm{W}}_{\mathrm{l}}^{\mathrm{n}}\mathrm{\varphi }\left(\sum_{\mathrm{i}=1}^{\mathrm{n}}{\mathrm{W}}_{\mathrm{li}}^{\mathrm{n}}{\mathrm{X}}_{\mathrm{i}}\right)\right]$$

13 $$\mathrm{O}={\mathrm{\varphi }}_{\mathrm{i}}\left(\sum_{\mathrm{i}=1}^{\mathrm{n}}{\mathrm{W}}_{\mathrm{i}}^{\mathrm{n}}{\mathrm{X}}_{\mathrm{i}}\right)+{\mathrm{\varphi }}_{\mathrm{o}}\left[{\mathrm{B}}^{\mathrm{k}}+\sum_{\mathrm{l}=1}^{\mathrm{k}}{\mathrm{W}}_{\mathrm{l}}^{\mathrm{n}}\mathrm{\varphi }\left({\mathrm{B}}^{\mathrm{n}}+\sum_{\mathrm{i}=1}^{\mathrm{n}}{\mathrm{W}}_{\mathrm{li}}^{\mathrm{n}}{\mathrm{X}}_{\mathrm{i}}\right)\right]$$

where: O is the output, W is the synaptic weight, X is the input signal, B is the bias, ${\mathrm{\varphi }}_{\mathrm{i}}$ is the activation function from the input layer to the output layer, ${\mathrm{\varphi }}_{\mathrm{o}}$ is the activation function from the hidden layer to the output layer, and $\mathrm{\varphi }$ is the activation function from the input layer to the hidden layer.

### Elman neural network

**Figure 5:**
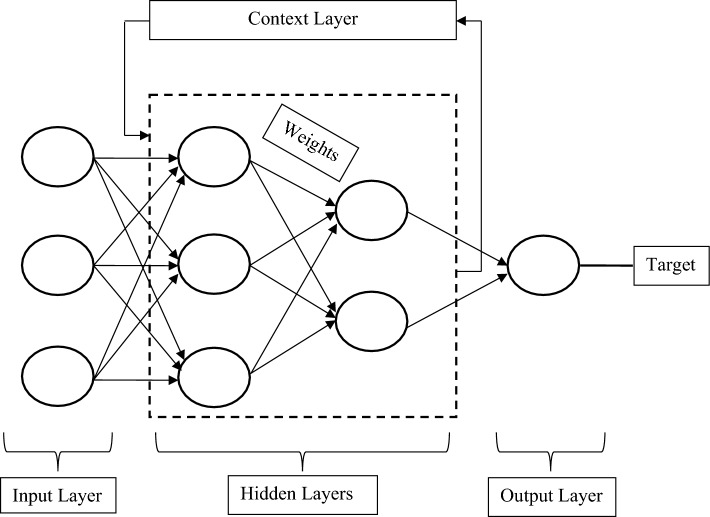
The basic architecture of the Elman neural network model.

Elman net is a recurrent type of neural network that was designed to recognize and predict the learned values or events^39^. This neural network mainly consists of an input layer, hidden layer, context layer, and output layer as shown in Fig. 5. Like a conventional multilayer perceptron, Elman net also has connections among the input layer, hidden layer, and output layer; however, an additional “context layer” is added to its architecture. The outputs of the hidden layer are fed to the context layer as input signals. The context layer processes these input signals, stores the values from the previous time step, and feeds them forward to the hidden layer as the input signals. In the case of any delay, this time cycle uses the previously stored values in the current time step, which reduces the overall computational time. According to Elman 1990^40^, the outputs of both the input layer and the context layer activate the hidden layer and then the outputs of the hidden layer are transmitted to the output layer. The hidden layer output units also activate the context layer. The output signals are compared with the teacher input signals and their estimated residuals are fed back for the adjustment of their synaptic weights. In the Elman net, the nonlinear sigmoid transfer function is applied to the hidden layer. While the output layer uses a linear purelin transfer function. The nonlinear-linear combination of transfer functions enhances the performance of this recurrent neural network. There must be a suitable number of neurons in the hidden layers to fit the function properly. This recurrent connection makes the Elman net favorable to detecting and generating time-varying patterns.

## Particle swarm optimization (PSO)

Kennedy and Eberhart in 1995^41^ proposed the use of the Particle Swarm Optimization (PSO) algorithm to find an optimal solution. PSO works on the principles of the socio-biological behavior of birds in their flock^42, 43^. As each bird searches for food randomly and disseminates this information to other birds in the flock. The mutual collaboration among the birds comes up with the best hunt. The same scenario can be simulated to find the best solution in a multidimensional space. Being a metaheuristic algorithm, the PSO tries to find the best global optimal that is very close to the real global optimal^44–46^.

In a population of P particles, the position, and velocity of a jth particle at iteration i can be expressed as follows:

14 $${X}^{j}\left(i\right)=\left[{x}^{j}\left(i\right), {y}^{j}\left(i\right)\right]$$

15 $${U}^{j}\left(i\right)=\left[{u}_{x}^{j}\left(i\right), {u}_{y}^{j}\left(i\right)\right]$$

The position and velocities of each particle update at the next iteration

16 $${x}^{j}\left(i+1\right)=\left[{x}^{j}\left(i\right), {u}_{x}^{j}\left(i+1\right)\right]$$

17 $${y}^{j}\left(i+1\right)=\left[{y}^{j}\left(i\right), {u}_{y}^{j}\left(i+1\right)\right]$$

18 $${U}^{j}\left(i+1\right)=w{U}^{j}\left(i\right)+{c}_{1}{r}_{1}\left[{pbest}^{j}-{X}^{j}\left(i\right)\right]+{c}_{2}{r}_{2}\left[gbest-{X}^{j}\left(i\right)\right]$$

where: U is the particle velocity, w is the inertial weight whose value is chosen between 0 and 1, c_1_ and c_2_ are cognitive and social coefficients, r_1_ and r_2_ are the random numbers between 0 to 1, pbest is the best position of a particle at given function and gbest is the best position of other particles in the swarm.

## Results and discussion

### The behavior of rocks subjected to the excitation frequencies

To investigate the dynamic behavior of rocks, samples were tested non-destructively at their ambient conditions under a set of excitation frequencies ranging from 7 to 16 kHz. The intact rock samples may have hidden flaws, microcracks, internal defects, etc.^47^. The propagation of high-frequency stress waves through rock samples causes the plastic deformation of microcracks. Such alterations can make a stiffer rock weaker and vice versa^48^.

**Figure 6:**
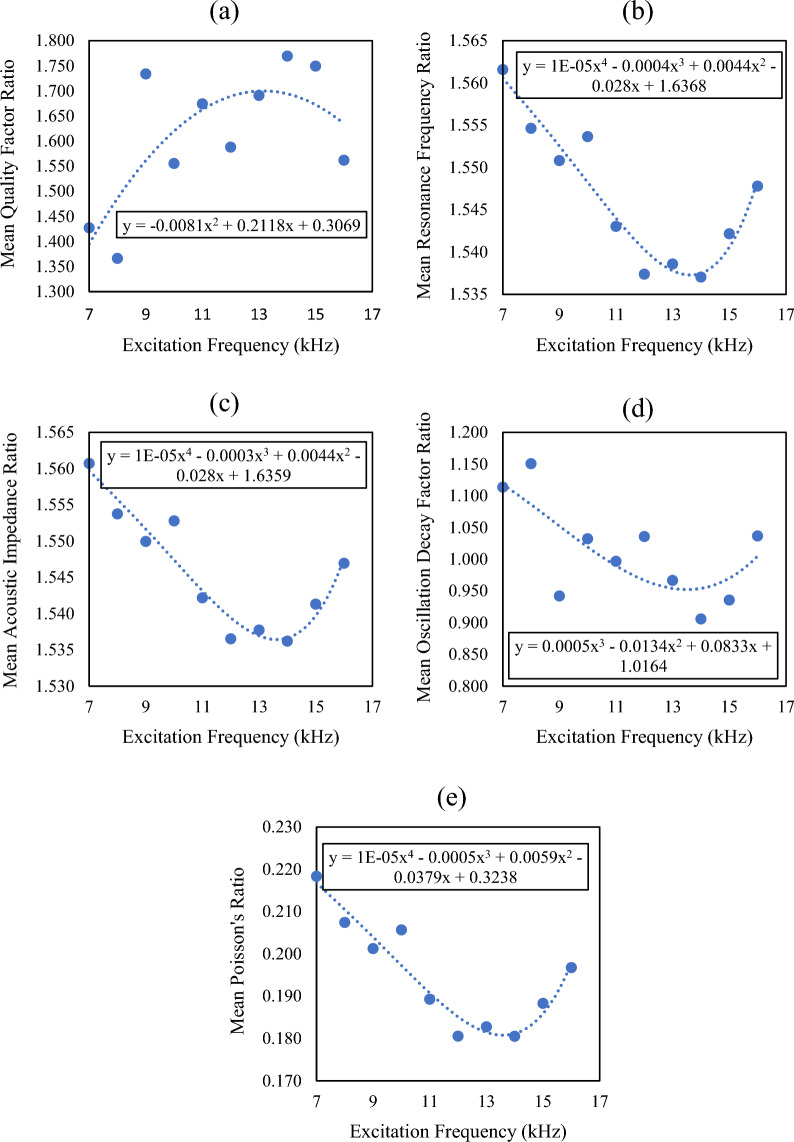
Variations in mean ratio factor values against excitation frequencies.

In this study rock samples were tested in both longitudinal and torsion modes. To anticipate the overall dynamic behavior of rocks, a ratio factor was determined by using the parameters measured in the longitudinal and torsion modes. Figure 6 shows the variations in the mean ratio factor values against increasing excitation frequencies. The ratio factor was determined in terms of the quality factor ratio (Q_r_), resonance frequency ratio (FR_r_), acoustic impedance ratio (Z_r_), oscillation decay factor ratio (α_r_), and dynamic Poisson’s ratio (*v*). The quality factor is a dimensionless parameter that describes the compactness of the rock. Figure 6a illustrates that the quality factor increases up to a certain level and then starts to decrease. This behavior signifies that an increasing excitation frequency produces the plastic deformation of microcracks, and the rock becomes stiffer. After getting a peak value, new microcracks start to develop that significantly affect the stiffness of the rock. Consequently, the quality factor declines.

Resonance is a phenomenon in which a material’s frequency synchronizes with the applied frequency^6^. Thus, the material’s particles vibrate with greater amplitude. In a stiffer material, particles have less chance to vibrate with greater amplitude as compared to the loose material. Figure 6b demonstrates that resonance frequency depreciates to its peak value and then starts to increase. The possible reason for this behavior is the plastic deformation of pre-existing cracks that enhances the rock stiffness. The acoustic impedance and oscillation decay factor can be elucidated in the same manner. Both parameters describe the soundness of the rock. A shattered or internally disrupted rock would have a lag time for stress wave propagation^49^. Therefore, Fig. 6c and d show a decreasing trend for acoustic impedance and oscillation decay factor respectively. Such depreciation and appreciation trends are owed to the plastic deformation of cracks and the development of new microcracks respectively.

Poisson’s ratio is the ratio of the transverse strain to the longitudinal strain. Figure 6e shows that the Poisson’s ratio decreases as the stiffness of the material increases^50^. The plastic deformation reduces the transverse strain more as compared to the longitudinal strain. Therefore, the entire fraction diminishes and leads to a reduction in Poisson's ratio. These results were noted only for the selected carbonate and silicate rocks. However, there is no guarantee that similar kind of results would be observed in other rocks. Because each rock type is composed of different minerals and exhibits different behavior under excitation frequencies.

### Correlation and sensitivity analysis

Table 3 shows the bivariate correlation between the measured parameters. The negative sign implies a downtrend and vice versa. Poisson’s ratio as a dependent variable did not make a strong correlation with the variables. Correlation values signify that the dimensionality issue would not affect the model performance. A bivariate correlation in terms of Pearson coefficient (R) above 0.5 is taken seriously and a strong correlation leads to the overfitting of the model^16^. Poisson’s ratio correlation value with the parameters ranged from − 0.19 to 0.16. Whereas, resonance frequencies and quality factors made slightly high correlations with each other and their values varied from 0.24 to 0.68. Oscillation decay factors and impedances had very strong correlations with one another ranging from 0.89 to 0.99. Neural network based models can easily accommodate highly correlated parameters without affecting model performance. That’s why it has a competitive edge over multi-linear regression modeling. However, in this study, to avoid dimensionality and overfitting issues, the same parameters measured in longitudinal and torsion modes were divided to get unitless factor ratios. These ratio parameters were regressed with Poisson’s ratio using the neural network modeling.

**Table 3:** Bivariate analysis between parameters.

	Q_p_	Q_s_	RF_p_	RF_s_	α_p_	α_s_	Z_p_	Z_s_	*v*
Q_p_	1.00								
Q_s_	0.42	1.00							
RF_p_	0.68	0.24	1.00						
RF_s_	0.66	0.25	1.00	1.00					
α_p_	− 0.43	− 0.28	0.36	0.37	1.00				
α_s_	0.04	− 0.76	0.43	0.43	0.50	1.00			
Z_p_	0.54	0.30	0.90	0.91	0.40	0.31	1.00		
Z_s_	0.53	0.30	0.89	0.91	0.40	0.31	0.99	1.00	
*v*	− 0.11	− 0.19	− 0.02	− 0.03	0.16	0.13	− 0.13	− 0.13	1.00

**Figure 7:**
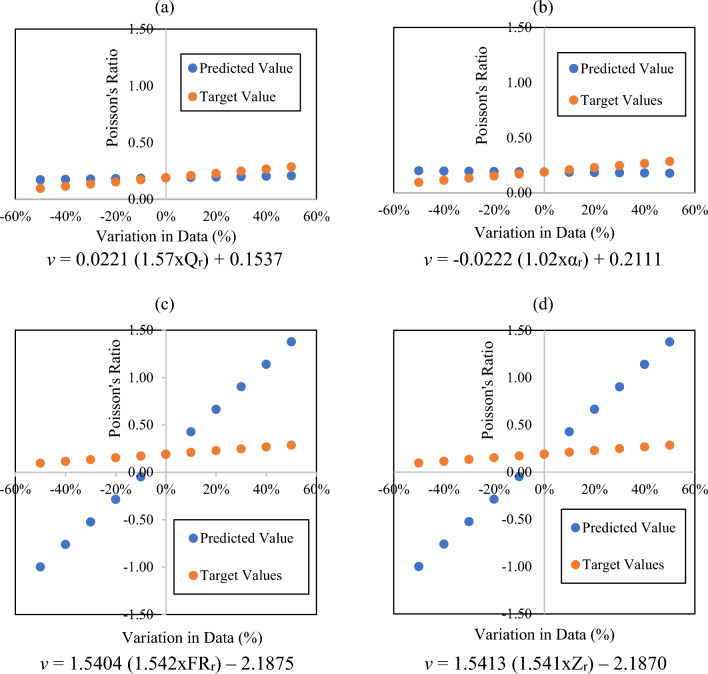
Sensitivity analysis to evaluate the degree of influence of regressors.

Sensitivity analysis was performed to find the degree of importance of input variables. In this technique, input variable values are varied to sensitize their influence on the target variable^51^. In linear-nonlinear modeling, it helps to select more robust parameters for regression. In this study, unitless factor ratios including quality factor ratio, resonance frequency ratio, impedance ratio, and oscillation decay factor ratio were regressed with the Poisson’s ratio in terms of one to one relationship. Their model equations are described in Fig. 7. To evaluate the sensitivity of input variables, a ± 50% data variation with respect to mean value was incorporated in the regressors. Figure 7a and b show that the quality factor ratio (range = 0.17–0.21) and oscillation decay factor ratio (range = 0.18 to 0.20) respectively are less sensitive to data variation and have fewer differences in predicted and estimated values (range = 0.10–0.29). On the other hand, Fig. 7c and d illustrate that the resonance frequency ratio (range = − 1.00–1.38) and impedance ratio (− 1.11 to 1.36) are more sensitive to data variability. Even only ± 10% of data variation leads to high residual errors between predicted and estimated values (range = 0.10–0.29).

### Data modeling and optimization

**Figure 8:**
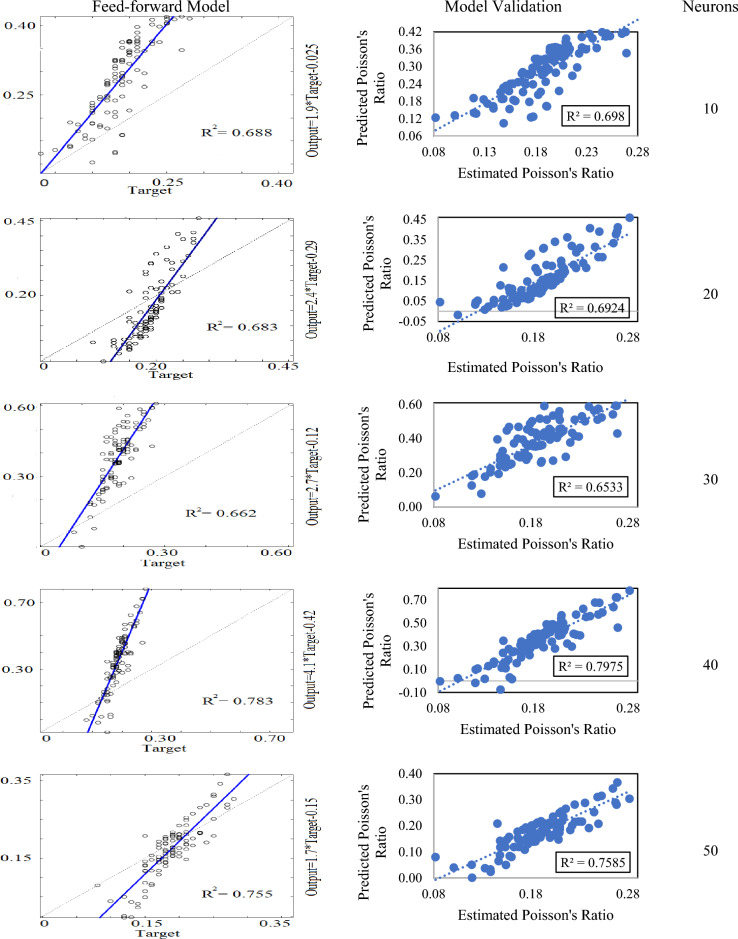
Feed-forward neural network models and their validation.

Apart from traditional linear-nonlinear regression analysis, this study focuses on using the artificial neural network for prediction modeling. For this purpose, backpropagation neural network algorithms including feed-forward, cascade-forward, and Elman were employed for the estimation of dynamic Poisson’s ratio. Overall, 15 neural network models were developed and one of them was selected for optimization. The number of neurons significantly plays an important role in developing the best fit model. A suitable number of neurons can be selected based on the hit-trial method. In the case of the feed-forward neural network, 5 models were developed by taking the number of neurons from 10 to 50. The feed-forward model with 40 neurons was considered the best one with a coefficient of determination (R^2^) value of 0.783. The rest of the models had a comparatively lower value of R^2^ ranging from 0.662 to 0.783 (see Fig. 8). The validation of these models by independent data also proposed the model with 40 neurons having a correlation coefficient (R^2^) value of 0.797. Whereas, the rest of the models had their R^2^ value from 0.692 to 0.758.

**Figure 9:**
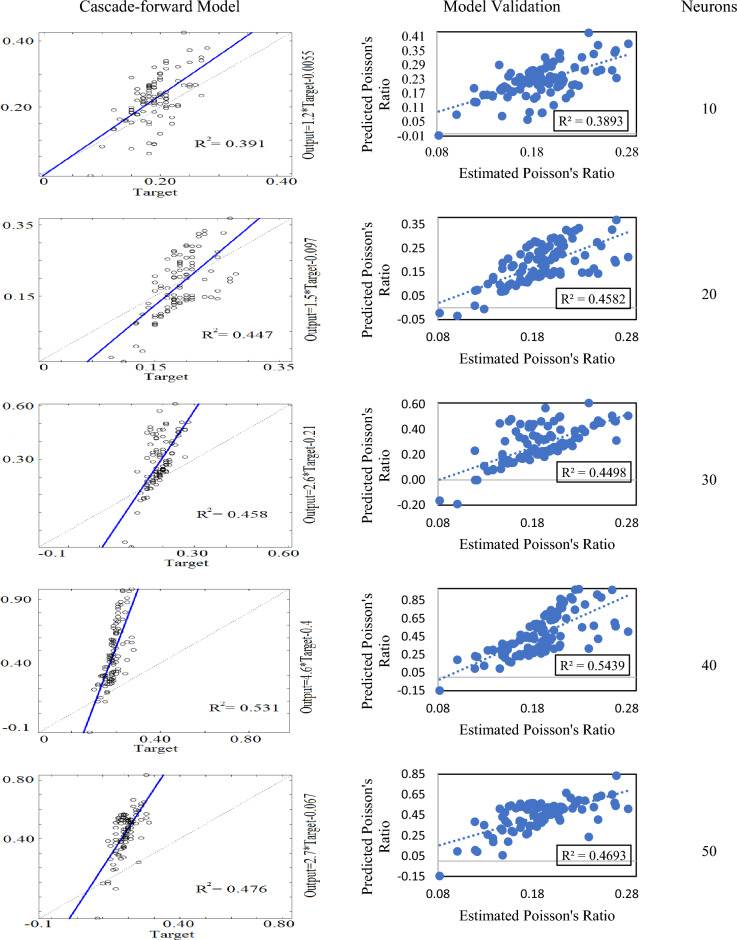
Cascade-forward neural network models and their validation.

In the case of the cascade-forward neural network, 5 models were developed by varying the number of neurons from 10 to 50. Among all developed models, a cascade-forward model with 40 neurons performed best and had the highest value of R^2^ (i.e. 0.531). Others showed a variation in their R^2^ values ranging from 0.391 to 0.531. All the models were validated by the experimentally acquired data and the model with 40 neurons had the highest value of correlation coefficient (i.e. R^2^ = 0.543) as compared to its counterparts. The validation results of the cascade-forward neural network models were found less significant than the outcomes of the feed-forward neural network models. As illustrated in Fig. 9, the variation in their R^2^ values was noted from 0.389 to 0.543.

**Figure 10:**
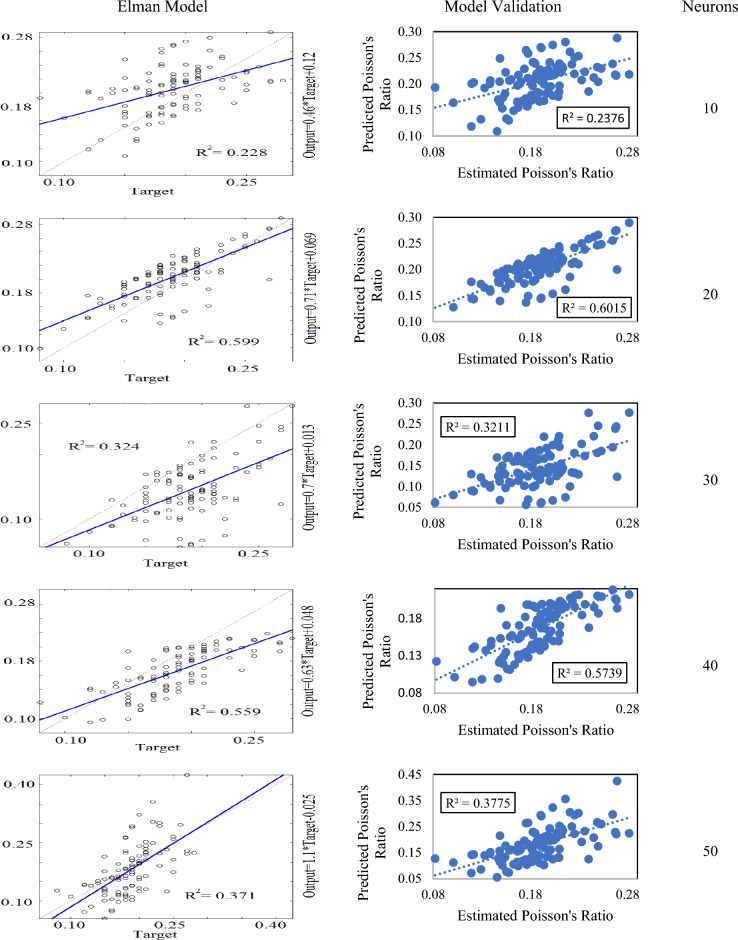
Elman neural network models and their validation.

For Elman neural network, 5 models were developed at a varying range of neurons from 10 to 50. Unlike feed-forward and cascade-forward models, the Elman model with 20 neurons had the highest value of R^2^ = 0.599. Whereas, others showed their determination coefficient values from 0.228 to 0.599. An independent dataset was used for the validation of these models. Results showed that the model with 20 neurons had a comparatively higher value of R^2^ (i.e. R^2^ = 0.601) than the rest of the models. The R^2^ of the remaining Elman models was found by 0.237 to 0.573 as shown in Fig. 10.

**Figure 11:**
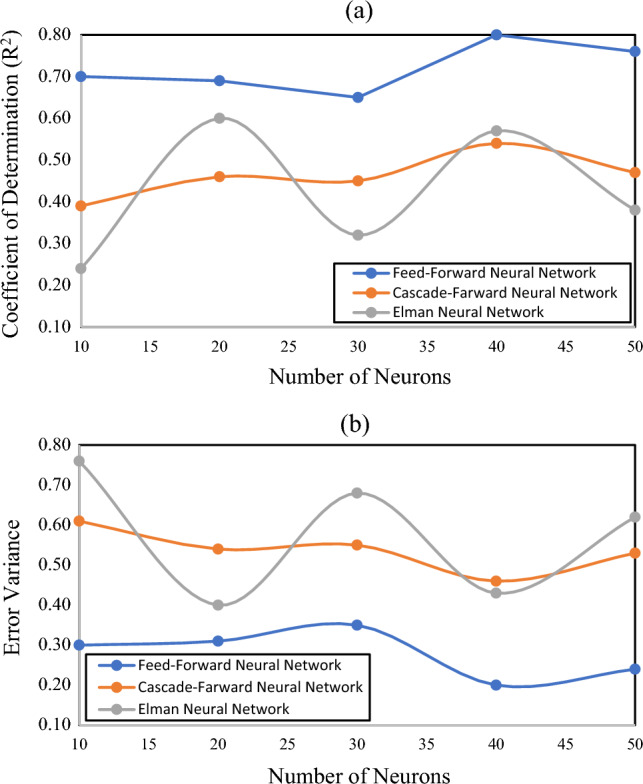
Relationship of model R^2^ and error variance with the number of neurons.

It is evident from the above discussion that the feed-forward algorithm produced better outcomes than the cascade-forward and Elman algorithms. Based on the model validation results, the feed-forward model with 40 neurons was considered the best one and optimized further to get a high-quality end product (see Fig. 11a). Figure 11b shows the relationship between the error variance and neurons. At each instance error variance in feed-forward models was noted as a minimum. However, in the case of cascade-forward and Elman models, a slight fluctuation in error variance was observed against the increasing number of neurons. Minimum error variance was found in cascade-forward models at 10, 30, and 50 neurons. Whereas, Elman models showed lower error variance at 20 and 40 neurons. This aspect indicates that the number of neurons considerably affects the model performance, and they must be selected after rigorous analysis.

**Figure 12:**
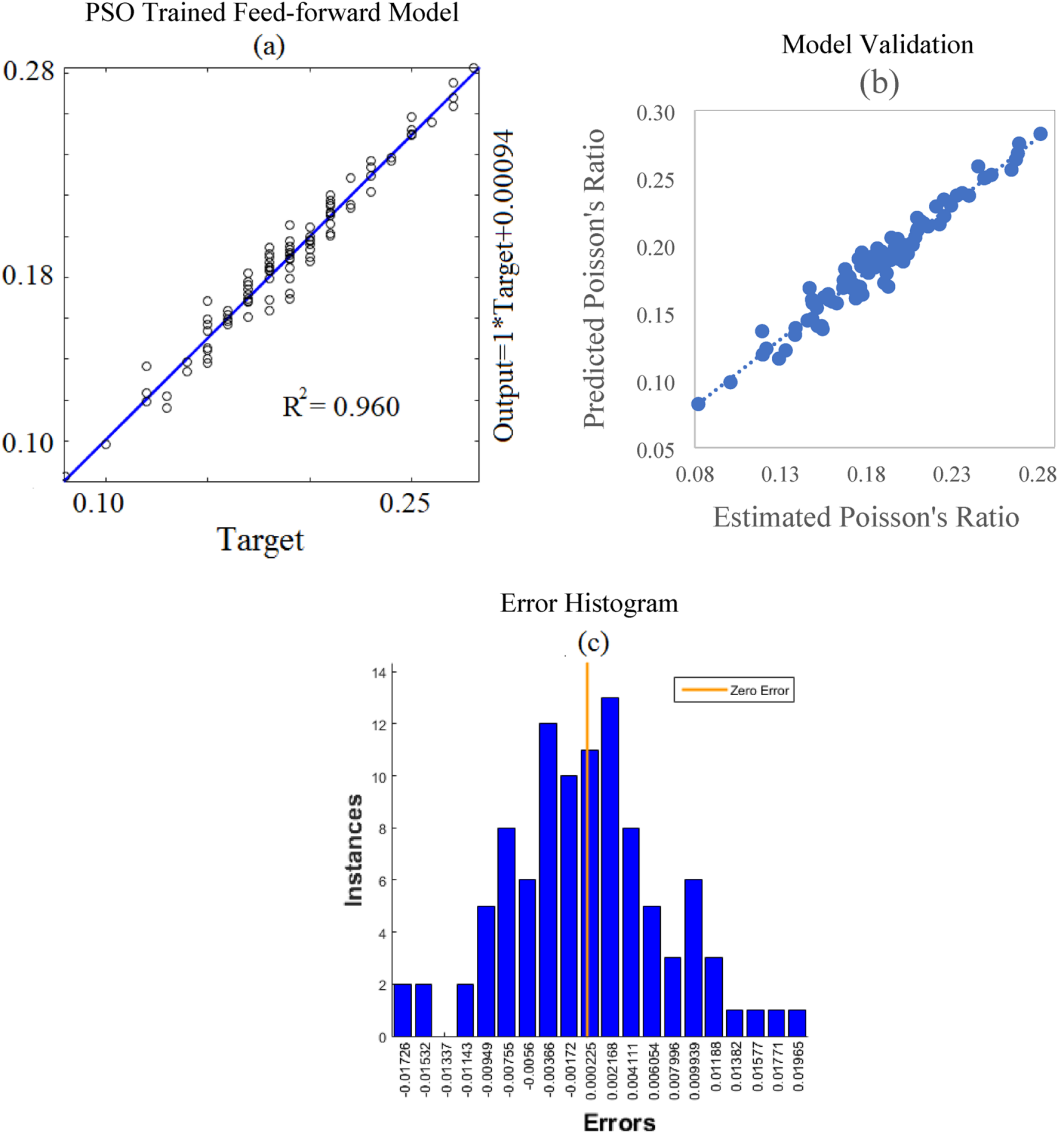
PSO trained neural network model and its validation along with the error histogram.

Among all backpropagation algorithms, the feed-forward model with 40 neurons was chosen for optimization because of its best performance. The optimization was carried out using the particle swarm optimization algorithm. PSO has a competitive edge over traditional optimizers due to its simplicity, ease of implementation, robustness, and computational accuracy. To use PSO the values of the cognitive and social coefficients were set as 1.5 and 2.5 respectively. Whereas, inertial weight and random numbers were selected between 0 to 1. Overall, 1000 iterations were carried along with the set of 50 swarms. Under the above-said conditions, the feed-forward model was trained to get a plausible model. Figure 12a shows that after the optimization the coefficient of determination value of the model was improved from 0.783 to 0.96. The model validation through an experimentally acquired dataset exhibited that the value of R^2^ got increased from 0.797 to 0.954 (see Fig. 12b). Figure 12c illustrates the error histogram of the model after optimization. It shows the error between predicted and target values. In this case, the bin size and instances were set at 20 and 14 respectively. The histogram shows the minimum error values against different instances which implies that the model was good enough for prediction.

## Conclusions

In this research, the dynamic behavior of rocks was investigated under ambient conditions. The overall variation in the values of Q_p_, Q_s_, FR_p_, FR_s_, Z_p_, Z_s_, α_p_, α_s_, and *v* was estimated by 20–28, 10–17, 1.56–2.68 kHz, 1.02–1.76 kHz, 9–20 MPa*sec/m, 6–13 MPa*sec/m, 0.24–0.33 kHz, 0.19–0.34 kHz, and 0.16–0.22 respectively. The mean ratio factor value of Q_r_, FR_r_, Z_r_, α_r_, and *v* was determined by 1.366–1.773, 1.524–1.562, 1.523–1.561, 0.906–1.612, and 0.160–0.218 respectively. The outcomes of the dynamic response of rocks reveal that the stiffness of rocks increases against the excitation frequencies and then starts to decrease due to the development of new microcracks.

After the evaluation of the dynamic behavior of rocks, prediction modeling was performed to estimate the dynamic Poisson’s ratio. It was regressed with the Q_r_, FR_r_, Z_r_, and α_r_ by using three backpropagation neural network algorithms including feed-forward, cascade-forward, and Elman. Overall, 15 models were developed by varying the number of neurons from 10 to 50. In the case of feed-forward and cascade-forward algorithms, the models with 40 neurons were found more plausible than the rest of the models. In the learning phase, the coefficient of determination for feed-forward and cascade-forward was estimated at 0.783 and 0.531 respectively. Whereas, during the validation stage, their coefficient of determination values were determined as 0.797 and 0.543 respectively. For Elman neural network algorithm, the model with 20 neurons was considered as best one. The coefficient of determination value for Elman net was calculated as 0.774. Among all models, the feed-forward model with 40 neurons comparatively performed much better. Therefore, a feed-forward net was chosen for the optimization to get a more robust model.

Results showed that the optimization of the selected model with the particle swarm optimization algorithm further improved its quality. After the training and validation of the model, its Pearson’s correlation coefficient and coefficient of determination values got increased from 0.885 to 0.980 and 0.797 to 0.954 respectively.

It is evident from the outcomes of this study that the optimization makes the neural network model more significant and robust. This approach can be used to solve several problems regarding data modeling. Furthermore, the results of rock behavior under dynamic cyclic loading can be utilized as a reference in the design work of mega-structures and to anticipate construction material response subjected to dynamic loadings.

## Data Availability

The datasets generated and/or analyzed during the current study are not publicly available due to some restrictions but are available from the corresponding author on reasonable request.
